# Food borne bacterial models for detection of benzo[a]pyrene‐DNA adducts formation using RAPD‐PCR

**DOI:** 10.1111/1751-7915.12355

**Published:** 2016-03-14

**Authors:** Valentina Lanzone, Rosanna Tofalo, Giuseppe Fasoli, Giorgia Perpetuini, Giovanna Suzzi, Manuel Sergi, Federica Corrado, Dario Compagnone

**Affiliations:** ^1^ Faculty of BioScience and Technology for Food, Agriculture and EnvironmentUniversity of TeramoMosciano Sant'Angelo (TE)64023Italy; ^2^ Istituto Zooprofilattico Sperimentale del MezzogiornoVia Salute 2, PorticiNapoli80055Italy

## Abstract

Random amplified polymorphic DNA (RAPD) PCR is a feasible method to evaluate genotoxin‐induced DNA damage and mutations. In this study, *Lactobacillus plantarum* ATCC 14917T, *Enterococcus faecium* DSMZ 20477T, *Escherichia coli* PQ37 and *Saccharomyces cerevisiae* S441 were screened for DNA genetic alterations by DNA fingerprinting using M13 and LA1 primers after treatment with three compounds forming covalent adducts with DNA [benzo[a]pyrenediol epoxide (BPDE), methyl methanesulfonate and 1,2,3,4‐diepoxybutane (DEB)]. M13 RAPD fingerprinting revealed that the total number of bands decreased in all treated DNA compared to control samples and generally the lost bands were characterized by high molecular weight. Some extra bands were detected for *L. plantarum* and *E. faecium*, while in *E. coli* and *S. cerevisiae* DNAs BPDE and DEB treatments did not result in new extra bands. Besides qualitatively analysis, cluster analysis based on Unweighted Pair‐Group Method with Average algorithm was performed to compare DNA fingerprints before and after treatments. This analysis confirmed the absence of significant differences between negative controls and treated DNA in *S. cerevisiae* and *E. coli* however the disappearance of some bands can be detected. The data indicate that this approach can be used for DNA damage detection and mutations induced by genotoxic compounds and highlighted the possible use of *L. plantarum* and *E. faecium* M13 based fingerprinting as reference for hazard identification in risk assessment.

## Introduction

Polycyclic aromatic hydrocarbons (PAHs) are well‐known toxic chemicals that must be strictly monitored because of their carcinogenic, mutagenic and teratogenic effect. They may be found in a variety of matrices including foods that can be contaminated from environmental sources, industrial food processing and from certain home cooking practices (Philips, 1999). Benzo[a]pyrene (BP) is the most toxic compound belonging to PAHs; it requires metabolic activation to reactive electrophiles for the covalent binding to DNA. Reactive metabolites with the most toxic effects have been identified as the different stereoisomers of benzo[a]‐pyrene 7,8‐dihydrodiol 9,10‐epoxide (BPDE) (Miller and Ramos, 2001). The covalent binding to DNA occurs almost exclusively at the exocyclic amino group of deoxyguanosine (dG), via trans or cis addition to the benzylic C‐10 position in the diol epoxide (Cosman *et al*., 1990). BP and other PAHs toxicity (or reactivity versus DNA) can be produced also via photochemical activation and different studies have been conducted have been conducted on the toxicity of PAHs exposed to UV and visible light. Photodegradation products have been demonstrated to be mutagenic, to induce a general damage to DNA (Shemer and Linden, 2007; Toyooka and Ibuki, 2007; Platt *et al*., 2008) and to form covalent adducts with guanine (Compagnone *et al*., 2011). There is a great concern on the effect of genotoxic compounds on the structure and function of DNA including adducts formation, DNA breakage and mutations. Rapid and sensitive methods to detect these types of alterations are thus needed.

Detection of DNA adducts is, however, a rather complex matter, because of the low frequency of DNA adduction that occurs *in vivo*. For this purpose, several highly sensitive techniques, which include immunoassays (Divi *et al*., 2002) fluorescence assays (Devanesan *et al*., 1996), ^32^P‐post labelling (Suzuki *et al*., 2004) and DNA‐based electrochemical or optical biosensors (Del Carlo *et al*., 2008; Wang *et al*., 2008; Lanzone *et al*., 2013) have been proposed.

Recently, advances in molecular biology have led to the development of several selective and sensitive assays, such as restriction fragment length polymorphism, quantitative traits loci, random amplified polymorphic DNA (RAPD), amplified fragment length polymorphisms, simple sequence repeat, variable number of tandem repeats for DNA analysis in eco‐genotoxicology (Cenkci *et al*., 2009). RAPD‐PCR is one of the most used technique for detection of DNA damage and mutations comparing DNA fingerprints from untreated and treated samples with genotoxic agents (Castano and Becerril, 2004; Theodorakis and Bickham, 2004; Liu *et al*., 2005; Atienzar and Jha, 2006; Enan, 2006).

Generally, treated samples are characterized by the appearance and disappearance of bands in comparison to control patterns (Atienzar and Jha, 2006). These alterations are associated to the presence of DNA adducts, mutations or DNA strand breaks (Atienzar and Jha, 2006). Some modifications of bands intensity can be also observed since large rearrangements can also occur when chemicals interact with genomic DNA. RAPD‐PCR offers several advantages. It does not require any previous knowledge about genome sequence and also avoids the use of radioisotopes. In addition, it is characterized by a high sensitivity and reliability and it is cheap since it is not requires specialized and expensive equipment (Atienzar and Jha, 2006). This technique is widely applied in different areas of research including clinical medicine, forensic science, pathogen detection, genotoxicants detection, genetically modified organisms, etc. It has been also used to detect genetic instability in tumours (Ong *et al*., 1998; Maeda *et al*., 1999; Yoke‐Kqueen and Radu, 2006) and DNA alterations induced by toxic compounds such as benzo[a]pyrene (Savva *et al*., 1994; Atienzar *et al*., 2002) in animals, bacteria and plants (Atienzar *et al*., 2002). However, the RAPD assay provides qualitative results since the nature and extent of DNA alterations can only be speculated unless the changes occurring in RAPD profiles are specifically analysed (e.g. cloning, sequencing, probing). Generally, model microrganisms used for the detection of DNA damage and mutations are *Saccharomyces cerevisiae* for yeasts (Frassinetti *et al*., 2011) and *Escherichia coli* for bacteria (Atienzar *et al*., 2002). To detect novel model microorganisms that can help the risk assessment of the genotoxic compounds, further studies focused on different model bacterial species seems necessary. This will help in understanding the effect of the compounds on different species and possibly in the selection of appropriate microorganism associated particularly to the food supply chain.

In this study, *Lactobacillus plantarum* ATCC 14917T, *Enterococcus faecium* DSMZ 20477T, *E. coli* PQ37 and *S. cerevisiae* S441 were screened for DNA genetic alterations by DNA fingerprinting using M13 and LA1 primers after treatment with three toxic compounds (BPDE, methyl methanesulfonate (MMS) and 1,2,3,4‐diepoxybutane (DEB)).

## Results and discussion

The RAPD technique is clearly a powerful tool to detect DNA damage (Enan, 2006). Aksakal and Esim (2015) proposed that RAPD PCR may potentially form the basis of novel biomarker assay to detect DNA damage and mutational events in bacteria, plants and animals. In fact, modified RAPD profiles as potential biomarkers has been successfully used to detect various types of DNA damage and mutations in animals, bacteria and plants induced by adverse conditions in environmental monitoring (Wong *et al*., 2000; Liu *et al*., 2005, 2007, 2009a,b; Aksakal *et al*., 2013; Aksakal and Esim, 2015). In this study, this approach was applied to detect DNA‐adducts formation in three different genomic DNA isolated from eukaryotic (*S. cerevisiae*) and prokaryotic (*L. plantarum* and *E. faecium*) microorganisms treated with three toxic compounds. *S. cerevisiae* was selected because is used as reference organism for genotoxicity tests, since it shows very simple growth conditions and short reproduction times (Frassinetti *et al*., 2011). In particular, *Saccharomyces cerevisiae* S441 was used as starter culture for Montepulciano d'Abruzzo Colline Teramane DOCG wine fermentation and has been demonstrated to exert an anti‐genotoxic and anti‐mutagenic action towards diphenyl‐1‐picrylhydrazyl, and to model genotoxins, 4‐nitroquinoline‐1‐oxide and *N*‐methyl‐*N*′‐nitro‐*N*‐nitrosoguanidine (Trotta *et al*., 2012). In addition, this strain presented acid‐bile tolerance showing similar behaviour to that of the probiotic strain *S. boulardii*, indicating that it could reach gut in viable form and thus prevent genotoxin DNA damage *in situ* (Trotta *et al*., 2012).

The selected bacteria were *E. faecium* and *L. plantarum*. *E. faecium* is a multifaceted lactic acid bacterium with an intimate relationship with human health and disease. It is able to colonize the gastrointestinal tracts and for most people, it is part of the normal commensal intestinal microbioma (Candela *et al*., 2010); it is also associated to several traditional foods (Giraffa, 2006). *L. plantarum*, is a highly versatile species found in several ecological niches (Siezen *et al*., 2010). It is able to survive gastric transit, and colonize the intestinal tract of human and other mammals (Vries *et al*., 2006). *E. coli* was used since it is considered a model for eco‐genotoxycological studies applying RAPD‐PCR (Atienzar *et al*., 2002).

The selection of appropriate primers represent a fundamental step in the development of RAPD‐PCR methodology. Different primers were screened to generate complex and informative RAPD profiles for both bacteria and yeast strains (data not shown). M13 and LA1 were eventually selected as they produced consistent and reproducible RAPD banding patterns in all tested species. M13 primer has been already used to highlight the genetic variability in different autochthonous strains isolated from several food ecosystems (Andrighetto *et al*., 2002; Tofalo *et al*., 2013, 2014). LA1 primer was designed on *S*. *cerevisiae* intron splice site and contains the lariat branch consensus sequence (TACTAAC), which is strictly conserved in the yeast *S. cerevisiae* as mutations in this site prevents splice of assembly and cleavage of the 5′ intron junction (Barros Lopes *et al*., 1996). This primer is widely used to type *S. cerevisiae* and non‐*Saccharomyces* wine strains.

**Figure 1:**
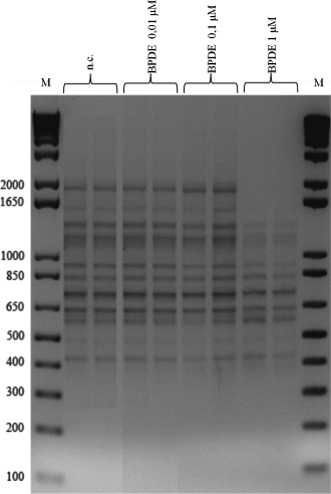
RAPD fingerprinting for *Saccharomyces cerevisiae* S441 strain with LA1 primer obtained for untreated and treated DNA samples using different concentrations of BPDE (0.01–0.1–1 μM). M: 1 kb DNA Plus ladder (Life Technologies (Monza, Italy)), n.c.: negative control.

The activated form of benzo(a)pyrene (BPDE) was used to test the reactivity versus DNA. LA1 primer was used to investigate the effects on RAPD profile of *S. cerevisiae* treated at three different concentrations of BPDE (0.01–0.1–1 μM). Using low concentrations of BPDE (0.01–0.1 μM) LA1 fingerprintings of treated DNA and negative controls were similar (Fig. 1). Minor modifications were obtained when the concentration was 1 μM; in particular, bands with higher molecular weight disappeared. Similar results were obtained for bacteria suggesting that BDPE acts in a dose dependent manner (data not shown).

More informative profiles for all concentrations tested were obtained using primer M13. This difference in sensitivity depending on primer sequence suggests a specificity in the mode of action of BDPE. The specificity of the mechanism of action of this chemical with hot spots in the DNA sequence, has been extensively supported in the literature (Castano and Becerril, 2004).

The principal events observed following the exposure to BDPE were a variation in the appearance (extra bands) and disappearance (band loss) of the amplified bands in the treated profiles in comparison to control profiles. The same effect was observed for MMS and DEB‐treated DNA. The number and molecular size (base pair, bp) of bands in untreated controls, BPDE, MMS and DEB‐treated DNA are summarized in Table 2. It should be emphasized that BPDE concentration in treated samples was lower (10^−3^) than those of the two other toxic compounds because of its greater reactivity (Laws *et al*., 2001).

The total number of bands decreased in treated DNA compared to control samples. In *E. faecium*,* L. plantarum* and *S. cerevisiae* some bands disappeared after DNA exposure to toxic compounds at the different concentrations tested (Table 1). Generally, the lost bands were characterized by high molecular weight. In particular, *E. faecium* RAPD profile exhibited the disappearance of seven bands with molecular size >1000 bp while *L. plantarum* and *S. cerevisiae* strains lost bands with a molecular weight ranging from 500 to over 1000 bp. The number of disappeared RAPD bands was greater with MMS treatments than BPDE and DEB. The disappearance of PCR products mainly affected the high molecular weight bands probably because the odds of obtaining DNA adducts increase with the length of the amplified fragment. Band loss may not only be related to different types of DNA damage (e.g. single‐ and double‐strand breaks, modified and oxidized bases, bulky adducts, DNA–protein cross links), point mutations, but also complex chromosomal rearrangements induced by genotoxins (Atienzar *et al*., 2002); in this case, it should be attributed to the formation of bulky adducts by all the genotoxic compounds.

**Table 1:** Final DNA and toxic compounds concentrations for all tested strains

DNA samples		Toxic compounds		
Genomic DNA	(ng ml^−1^)	BPDE (μM)	MMS (mM)	DEB (mM)
*Enterococcus faecium*	20	4	4	4
*Escherichia coli*	20	4	4	4
*Lactobacillus plantarum*	30	6	6	6
*Saccharomyces cerevisiae*	15	3	3	3

Some extra bands were also observed. In *E. faecium* treated DNA; the size of extra bands ranged from 100 to 1000 bp while in *L. plantarum* and *S. cerevisiae*, after treatment with MMS, the range was 100–500 bp. In *S. cerevisiae* DNA, BPDE and DEB treatments did not result in new extra bands. To detect new PCR product visible in agarose gel a rate of mutation ranging from 2% to 10% is required (Atienzar *et al*., 2002; Atienzar and Jha, 2006). For example, ‘hot spot’ interactions between DNA and metabolized B(a)P products have been reported in the literature (Boles and Hogan, 1984).

Extra bands are induced by mutations which could create new annealing events, by large deletions (bringing two pre‐existing annealing sites closer) and by homologous recombination, juxtaposing two sequences that match the sequence of the primer (Atienzar *et al*., 2000).

A reduction in the signal intensity of some bands in treated samples was also observed suggesting change in the number of copies of the sequence. Similar results have been recently obtained in Cd‐stressed *Arabidopsis thaliana* (Arabidopsis, Columbia ecotype) (Liu *et al*., 2012). In addition, it should be related to mutations altering the stability of the primer–template interaction without changing absolutely a primer's ability to anneal (Atienzar and Jha, 2006). Finally, in *S. cerevisiae*, a reduced intensity could be a result of aneuploidy, chromosome lost which induce a reduction in gene copy number.

**Figure 2:**
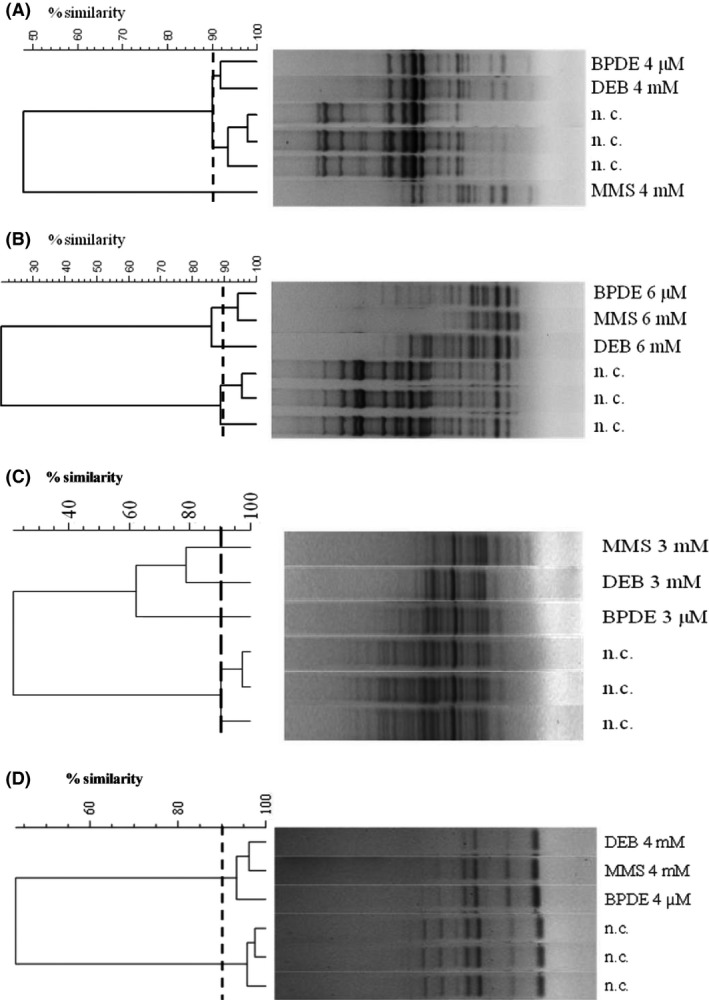
Dendrogram showing the similarity among RAPD‐PCR patterns of *Lactobacillus plantarum* ATCC 14917^T^ (A), *Enterococcus faecium* DSMZ 20477T (B), (C) *Saccharomyces cerevisiae* S441 and *Escherichia coli* PQ37 (D) after exposure to toxic compounds tested. Similarities were calculated using UPGMA. Arbitrary threshold 90% was used to identify new adducted biotypes. n.c.: negative control.

To better highlight these differences in signal intensity besides qualitatively analysis, based on disappearance and/or appearance of RAPD bands, Pearson correlation coefficient was used to construct dendrograms since this index does not consider just the presence/absence of the bands to evaluate the strain's differences, as the Dice or Jaccard indexes, but depends on the variance between two fluorescence density values at each point in the curve pattern and does not suffer from typical peak/shoulder mismatches (Seward *et al*., 1997). In fact, the band‐based Dice coefficient method is based on the comparison of designated band positions and divides the number of matching bands between patterns by the total number of bands, thereby emphasizing the matching bands (Dice, 1945). The Pearson correlation coefficient method compares the whole densitometric curves of patterns and is independent of band definition (Pearson, 1926). This approach resulted to be an effective method to establish the relationships among samples (Atienzar and Jha, 2006; Tofalo *et al*., 2014). DNA fingerprints before and after treatments were compared. Cluster analysis for *E. faecium*,* L. plantarum*,* S. cerevisiae* and *E. coli* is reported in Fig. 2.

For *E. faecium* DSMZ 20477T, using a similarity level of 90%, two main clusters were identified consisting in untreated negative controls and samples treated with BPDE and DEB. The DNA exposed to MMS clustered alone with a similarity degree lower than 50%. In particular, the main effects were the disappearance of bands with molecular weight between 850 and 2000 bp and the simultaneous appearance of amplification products ranging from 300 to 600 bp, as reported above.

Cluster analysis of *L*. *plantarum* revealed that DNA exposed to BPDE, MMS and to DEB formed two distinct clusters using a similarity level of 90%. In fact, BPDE and MMS treated DNA showed the same effects with the disappearance of amplifications products with molecular weight over 500 bp, while treatment with DEB did not cause effects on the amplification products ranging between 500 and 850 bp (Table 2).

**Table 2:** The number and size (bp) of extra and loss bands for M13‐RAPD profiles in treated and control samples

Range size (base pair)	*Enterococcus faecium* Untreated control	DNA treatments (mM)			*Lactobacillus plantarum* Untreated control	DNA treatments (mM)			*Saccharomyces cerevisiae* Untreated control	DNA treatments (mM)		
		BPDE (4 × 10^−3^)	MMS 4	DEB 4		BPDE (6 × 10^−3^)	MMS 6	DEB 6		BPDE (3 × 10^−3^)	MMS 3	DEB 3
>1000	9	2	2	2	8	3	0	3	7	4	1	2
500–1000	3	7	5	6	6	5	3	5	7	6	6	6
100–500	2	3	6	3	5	7	6	6	4	4	7	4
Total	14	12	13	11	19	15	9	14	18	14	14	12

For *S. cerevisiae* applying a similarity level of 90% DNA exposed to BPDE, MMS and to DEB formed three distinct clusters (Fig. 2). As reported above, the treatment with all tested compounds induced the disappearance of some high molecular weight bands. In particular after the treatment with MMS, some extra bands with a molecular weight ranging from 100 to 500 bp were detected (Table 2). However, the modifications were less evident than for bacteria (Fig. 2 and Table 2). In fact, bacteria RAPD profiles showed more differences in terms of disappearance and appearance of bands compared to *S. cerevisiae*. For this yeast, in fact, significant differences were obtained only after the treatment with MMS. It has been already reported that different regions of the genome are not equally sensitive to the toxic effects of these compounds, with regions less prone to modifications than others (Cambier *et al*., 2010).

In conclusion, the obtained results indicate that RAPD‐PCR can be used for the detection of DNA damage and mutations induced by BPDE, MMS and DEB with high sensitivity. The alterations were detected in as losses and/or gains of bands and variations in bands intensity. This approach allowed to show differences in the sensitivity of analysed food borne associated microorganisms to these compounds. In particular, bacteria were more sensitive than yeast to the treatment. For the first time, M13 fingerprinting of *L. plantarum* and *E. faecium* have been proposed as reference for hazard identification in risk assessment in eco‐genotoxicological studies, because even a low mutation rate can be identified. Further studies are necessary to better understand the mechanisms of DNA‐adducts formation.

## Experimental procedures

### Chemicals

Benzo[a]pyrenediol epoxide, was obtained by National Cancer Institute, Midwest Research Institute (Kansas City, MO, USA). MMS, DEB and all other reagents were from Sigma Aldrich, Italy.

### Bacterial and yeast strains and growth conditions

The strains used in this study were *E. faecium* DSMZ (Deutsche Sammlung von Mikroorganismen und Zellkulturen) 20477T, *L. plantarum* ATCC (American Type Culture Collection) 14917T, *E. coli* PQ37 and *Saccharomyces cerevisiae* S441, belonging to the Culture Collection of Faculty of BioScience and Technology for Food, Agriculture and Environment (University of Teramo, Italy) and isolated from Montepulciano d'Abruzzo wine producing area in Central Italy where the wine is produced based on spontaneous fermentation and without any commercial enzyme preparations. Bacteria and yeast were stored at −80°C in the Man Rogosa Sharpe (MRS) (Oxoid, Milan, Italy) (*E. faecium* and *L. plantarum*), in Luria Bertani (LB) medium (*E. coli*) and YPD medium (1% yeast extract, 2% peptone, 2% glucose), respectively, containing 20% (v/v) glycerol. Before experimental use, bacteria were propagated in MRS broth at 30°C in microaerophilic condition, whereas yeasts strains were grown in YPD for 16–18 h under aerobic conditions at 28°C.

### Genomic DNA isolation

Genomic DNA of *E. faecium* DSMZ 20477T, *L. plantarum* ATCC 14917T and *E. coli* were extracted according to de Los Reyes Gavilán *et al*. (1992) from 2 ml samples of overnight cultures grown anaerobically in MRS at 30°C. The final concentration of lysozyme used for cell lysis was 2 mg ml^−1^.

*Saccharomyces cerevisiae* S441 genomic DNA was extracted according to Aa *et al*. (2006) from 2.5 ml samples of overnight cultures grown at 30°C in YPD. Cell lysis was achieved using a lysis buffer (1% sodium dodecylsulfate, 5 mM NaCl, 10 mM Tris, 1 mM EDTA (Ethylenediaminetetraacetic acid), pH 8.0), chloroform, phenol (pH 6.6) and TE buffer (10 mM Tris, 1 mM EDTA). All obtained DNAs were resuspended in 30 μl TE buffer (10 mM Tris/HCl, 1 mM EDTA) pH 8.

Before treatment with toxic compounds, DNA concentration was estimated using the E‐Gel^®^ Low Range Quantitative DNA Ladder after electrophoretic migration on agarose gel (Life Technologies, Monza, Italy) in buffer TAE 1× (Tris‐acetate 0.04 M, EDTA 0.001 M, pH 8.3). Gels were stained with ethidium bromide 0.5 μg ml^−1^, washed with deionized water and photographed under UV transillumination using a Gel Doc 2000 EQ System (Bio‐Rad, Milan, Italy). DNA concentrations were quantified by comparing band relative intensities with the marker ones. The DNA quality was estimated by measuring the OD (optical density) at 260/280 nm in a Photometer (Amersham Pharmacia Biotech, Milan, Italy). For each sample, the DNA concentration was adjusted to 10 ng μl^−1^.

### DNA‐adducts formation

For adducts formation 5 μl of each DNA samples was diluted in 1× PCR buffer (Tris 20 mM, KCl 50 mM, pH 8.4) with 5 μl of toxic compound in a final volume of 50 μl. Final DNA and toxic compounds concentrations were reported in Table 1. Different concentrations of extracted genomic DNA led to different toxic compounds concentrations in the treatment (the ratio between DNA and toxic compound concentrations is the same for each DNA sample). The reaction was carried out for 24 h at 37°C and slow cooling at room temperature. Samples were stored at −20°C until use.

### RAPD fingerprints

RAPD‐PCR analysis was performed using the oligonucleotides M13 (5′‐GAGGGTGGCGGTTCT‐3′), an ubiquitous microsatellite sequence and LA1 (5′‐GCGACGGTGTACTAAC‐3′), an intron splice site primer. Amplification reactions were carried out on a Perkin‐Elmer GeneAmpPCR System 2400 (Milan, Italy). M13 amplification was performed according to Tofalo *et al*. (2014) with an initial denaturation at 94°C for 4 min followed by 35 cycles consisting of 30 s at 94°C, 20 s at 45°C, 2 min at 72°C and a final extension of 7 min at 72°C. LA1 amplification conditions were those described by Barros Lopes *et al*. (1996), except for the extension temperature of the amplification cycle (72°C instead of 74°C). In both cases, PCR products were separated on a 1.5% agarose gel in 1× TAE buffer using 1 kb Plus DNA (Life Technologies) as a marker.

After electrophoresis, the gels were stained with ethidium bromide 0.5 μg ml^−1^, washed with deionized water and photographed under UV transillumination using a Gel Doc 2000 EQ System. The repeatability of RAPD‐PCR fingerprints was determined by triplicate loading of independent triplicate reaction mixtures prepared with the same strain. Conversion, normalization, and further analysis of the RAPD‐PCR patterns were carried out with Fingerprinting II Informatix^™^ software program (Bio‐Rad). Similarities among profiles were calculated by using the Unweighted Pair‐Group Method with Average (UPGMA) algorithm. RAPD bands size estimation was assessed using Image Lab^™^ Software Version 3.0 (Bio‐Rad).
